# Clinical and Radiological Features of Unilateral Posterior Reversible Encephalopathy Syndrome (PRES): A Case Report

**DOI:** 10.7759/cureus.58774

**Published:** 2024-04-22

**Authors:** Seraj Makkawi, Osama Khojah, Salem K Baeshen, Muhammad E Ahmed

**Affiliations:** 1 College of Medicine, King Saud Bin Abdulaziz University for Health Sciences, Jeddah, SAU; 2 Research and Development, King Abdullah International Medical Research Center, Jeddah, SAU; 3 Department of Neurosciences, Ministry of the National Guard-Health Affairs, Jeddah, SAU; 4 Department of Neurology, King Fahad General Hospital, Jeddah, SAU; 5 College of Medicine, King Saud Bin Abdulaziz University for Health Sciences, Riyadh, SAU; 6 Department of Radiology, Ministry of the National Guard-Health Affairs, Jeddah, SAU

**Keywords:** unilateral posterior reversible encephalopathy syndrome, atypical posterior reversible encephalopathy syndrome, pres, reversible posterior leukoencephalopathy syndrome, posterior reversible encephalopathy syndrome

## Abstract

Posterior reversible encephalopathy syndrome (PRES) is a clinicoradiological entity characterized by reversible vasogenic edema predominantly affecting the posterior regions of the cerebral hemispheres. However, unilateral presentation of PRES is an exceptionally rare manifestation.

We describe the case of a 34-year-old woman who presented with left-sided hemiparesis, hemisensory loss, headache, and focal motor seizures. Brain CT revealed right anterior and posterior hypodensities in the right frontal and parietal subcortical locations, brain MRI showed vasogenic edema in the subcortical right cerebral hemisphere, and cerebral angiogram revealed diffuse narrowing of the left internal carotid artery just distal to the carotid bifurcation with no flow of contrast beyond the ophthalmic segment. The patient’s symptoms resolved during her admission, MRI findings improved on repeated imaging, and she was ultimately diagnosed with unilateral PRES.

Unilateral PRES is a complex and challenging diagnosis, and this case sheds light on the atypical radiological features of unilateral PRES possibly intricately linked with contralateral steno-occlusive disease of the carotid artery. It is essential to maintain the atypical variants of PRES as part of the differential diagnosis when encountering acute neurological symptoms and vasogenic edema on imaging in the context of contralateral steno-occlusive disease of the carotid artery.

## Introduction

Posterior reversible encephalopathy syndrome (PRES) is a clinicoradiological disorder characterized by a reversible disturbance of cerebral vasoreactivity, resulting in the development of characteristic neuroimaging findings [1]. PRES was first identified as a distinct imaging pattern among patients admitted for acute illnesses with symptoms such as headache, visual disturbances, seizures, and focal neurological deficits [2]. Subsequent literature on PRES has reported a wide variety of symptoms stemming from cerebral edema [3,4]. PRES typically occurs in the context of elevated blood pressure, exposure to certain medications (e.g. cyclosporine and tacrolimus), autoimmune diseases, and/or renal injury [5,6]. The precise underlying pathophysiology of PRES remains incompletely understood, although two proposed mechanisms have been discussed in the literature. The first hypothesis is related to elevated arterial blood pressure that exceeds cerebral autoregulation capacity, which leads to vasogenic edema due to dysfunction of the blood-brain barrier. The posterior circulation is particularly vulnerable to this process due to its relative lack of sympathetic innervation compared to the anterior circulation [5]. The second hypothesis involves endothelial dysfunction resulting from endogenous (preeclampsia, sepsis, and autoimmune disorders) or exogenous (chemotherapy and immunosuppressive agents) toxins [5].

Classically, PRES is considered a bilateral condition, but there have been occasional reports of unilateral involvement. The initial diagnosis of the first unilateral case of PRES, which was initially thought to be a neoplasm, underscores the diagnostic challenges associated with this condition [1]. However, difficulties in accurately diagnosing unilateral PRES have continued to occur in subsequent cases in the literature [7]. In this article, we aimed to describe the clinical presentation and radiological features of a patient with unilateral PRES, which can be linked to contralateral steno-occlusive disease of the carotid artery. ​​This case underscores the intricate interplay between chronic medical conditions, acute neurological events, and the critical importance of tailored, multidisciplinary care in managing complex cases of PRES.

## Case presentation

A 34-year-old woman presented to the emergency department with a new onset of pressure-like headache, left-sided weakness and numbness, and intermittent jerky movement of the left upper limb. Her medical history included type 1 diabetes, chronic hypertension, epilepsy, hypothyroidism, peripheral arterial disease, myelodysplastic syndrome, and end-stage renal disease requiring dialysis. She experienced focal motor seizures after being seizure-free for six years on a regimen of levetiracetam and valproic acid. Examination revealed hypertension reaching as high as 229/122 mmHg, distal left hemiparesis, and length-dependent sensory loss in the lower limbs reaching the mid-tibia. Cranial nerves, deep tendon reflexes, and coordination examinations were unremarkable.

**Figure 1:**
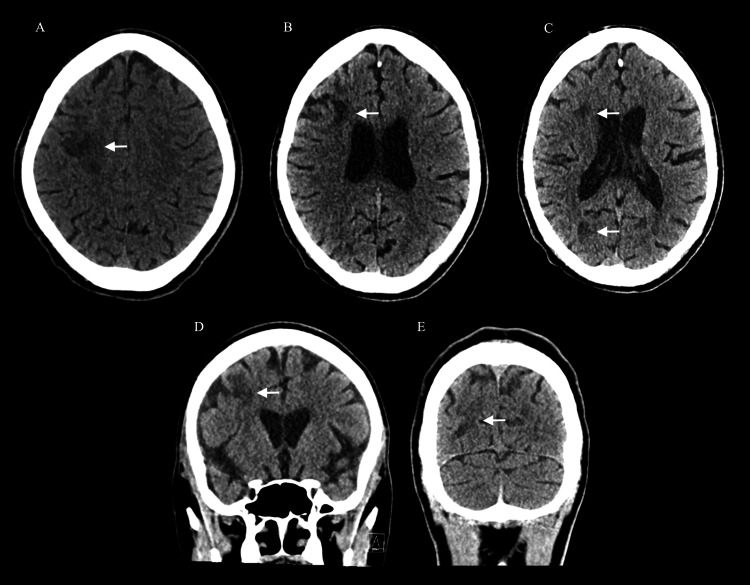
(A-C) Axial and (D, E) coronal brain CT without contrast performed on the first day demonstrating ill-defined subcortical frontal and parietal hypodensities (white arrows) Computed tomography (CT)

Initial laboratory investigations were unremarkable. Brain computed tomography (CT) revealed multiple patchy hypodensities involving right frontal and parietal subcortical locations (Figure 1).

The patient was admitted to the intensive care unit with a provisional diagnosis of a focal motor seizure with intact awareness and a subacute stroke. The patient was started on aspirin, received a loading dose of levetiracetam, and was managed with the maintenance of levetiracetam, valproic acid, and carbamazepine to achieve seizure freedom; valsartan, hydralazine, nifedipine, labetalol, and arranged for a dialysis session to achieve a blood pressure goal of <180/110 mmHg. These interventions were able to control her blood pressure and seizures.

An electroencephalogram was normal. Comprehensive testing for a stroke-in-young was conducted and included extensive vasculitis and thrombophilia work-up. Antinuclear antibody (ANA), anti-double-stranded DNA (dsDNA), cyclic citrullinated peptide (CCP) immunoglobulin G (IgG), anti-SS-A, anti-SS-B, cytoplasmic staining-antineutrophil cytoplasmic antibodies (c-ANCA), perinuclear/nuclear staining-antineutrophil cytoplasmic antibodies (p-ANCA), anti-SM, anticardiolipin antibodies (ACA) IgA, ACA IgM, ACA IgG, B2GP IgM, B2GP IgG, C3 complement, C4 complement, and protein S activity were all within normal limits. CSF analysis revealed normal WBC, RBC, and total protein levels but elevated glucose levels (Table 1).

**Table 1:** Results of the extensive vasculitis and thrombophilia workup and CSF analysis Antinuclear antibody (ANA); double-stranded DNA (dsDNA); cyclic citrullinated peptide (CCP); cytoplasmic staining - antineutrophil cytoplasmic antibodies (c-ANCA); perinuclear/nuclear staining - antineutrophil cytoplasmic antibodies (p-ANCA); anti-Smith (Sm) antibody; anticardiolipin antibodies (ACA); beta-2-glycoprotein (B2GP); cerebrospinal fluid (CSF); white blood cells (WBC); red blood cells (RBC)

Test	Patient’s result	Normal range
ANA	Negative	Negative
Anti-dsDNA	18.07 IU/mL	0-200 IU/mL
CCP IgG	3.26 U/mL	0-20 U/mL
Anti-SS-A	0.88 U	0-20 U
Anti-SS-B	1.65 U	0-20 U
c-ANCA	3.05 U	0-20 U
p-ANCA	1.95 U	0-20 U
Anti-Sm antibody	2.05 U	0-20 U
ACA IgA	1.28 APL U	0-12 APL U
ACA IgM	<0.1 MPL U	0-12 MPL U
ACA IgG	2.45 GPL U	0-12 GPL U
B2GP IgM	0.67 SMU	0-20 SMU
B2GP IgG	2.02 SGU	0-20 SGU
C3 complement	1.15 g/L	0.9-1.8 g/L
C4 complement	0.40 g/L	0.1-0.4 g/L
Protein S activity	88%	65-140%
CSF WBC	0 leukocytes/µL	0-5 leukocytes/µL
CSF RBC	0 cells/µL	0 cells /µL
CSF protein	0.41 mg/mL	0.15-0.6 mg/mL
CSF glucose	4.9 mmol/L	2.77-4.44 mmol/L

**Figure 2:**
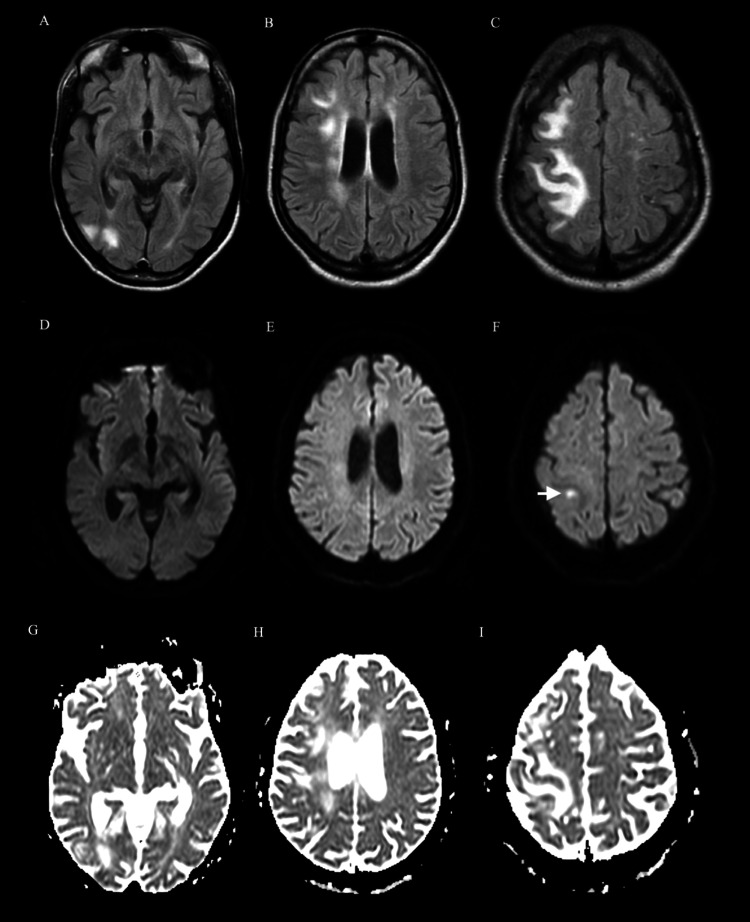
(A-I) Axial MR images performed on day three demonstrated vasogenic edema in the right cerebral hemisphere. (A-C) FLAIR sequence showing hyperintense lesions in the subcortical and deep white matter of the frontal, parietal, and occipital lobes of the right cerebral hemisphere with subtle involvement of the left hemisphere. (D-F) DWI sequence showing a tiny focus of restricted diffusion along the anterior aspect of the right post-central gyrus (white arrow). (G-I) ADC sequences showing patchy hyperintensity corresponding to the T2 shine-through effect consistent with vasogenic edema Magnetic resonance (MR); fluid-attenuated inversion recovery (FLAIR); apparent diffusion coefficient (ADC); diffusion-weighted imaging (DWI)

Brain magnetic resonance imaging (MRI) and magnetic resonance venography (MRV) were performed on day three and revealed vasogenic edema involving subcortical locations of the right frontal and parietal lobes (Figure 2), indicating PRES.

**Figure 3:**
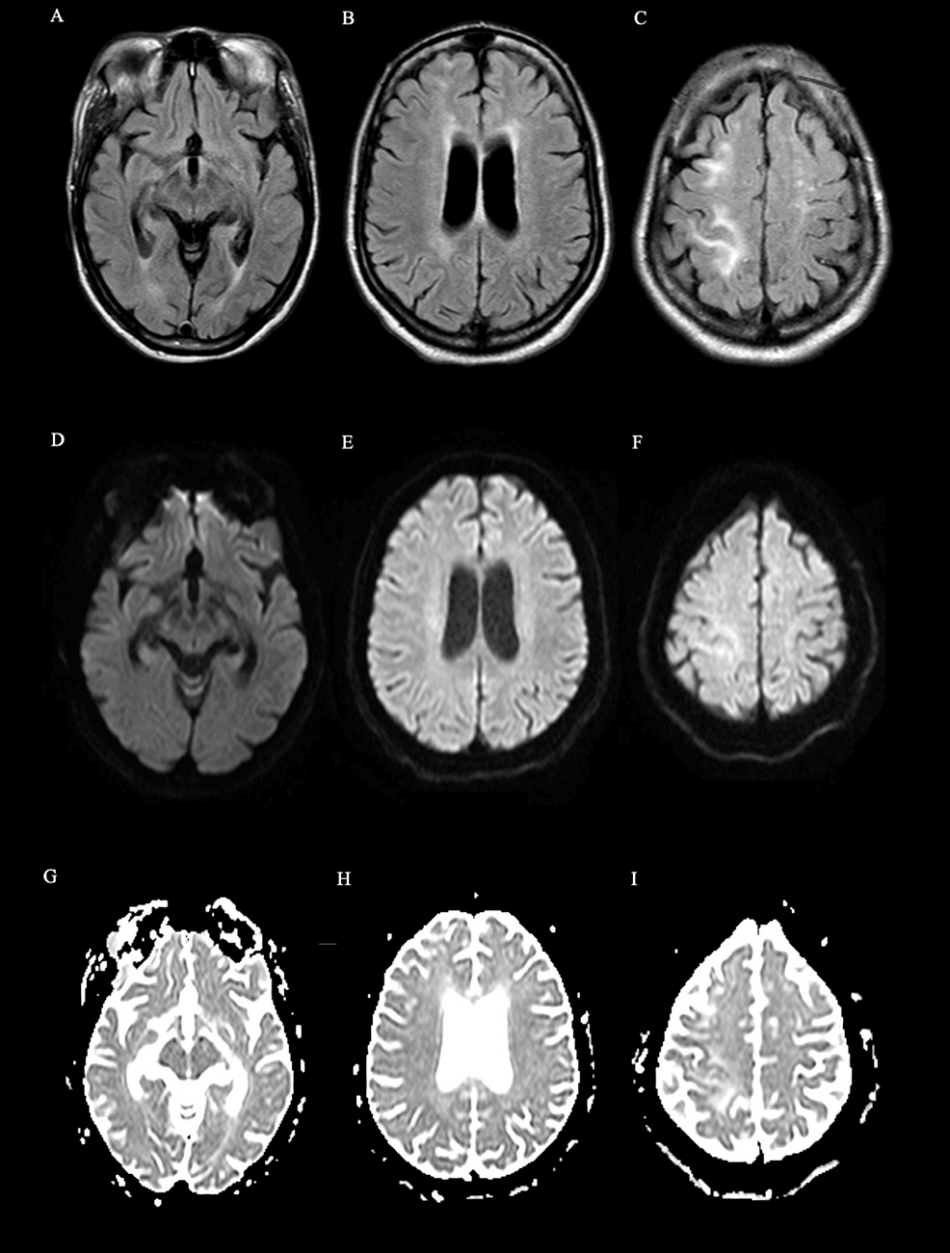
(A-I) Axial MR images performed on day nine demonstrated significant interval improvement of the vasogenic edema. (A-C) FLAIR sequence showing the interval improvement of hyperintense lesions seen on previous imaging. (D-F) DWI sequence showing interval resolution of previously seen focus of restricted diffusion. (G-I) ADC sequence showing interval improvement of hyperintense lesions seen on previous imaging. Magnetic resonance (MR); fluid-attenuated inversion recovery (FLAIR); apparent diffusion coefficient (ADC); diffusion-weighted imaging (DWI)

This prompted further investigation via CT angiography of the brain and neck, which was suboptimal due to contrast reflux in the cervical veins obscuring the bilateral carotid arteries. However, diffuse narrowing of the left internal carotid artery (ICA) was observed. This finding was supported by carotid ultrasound, which revealed left ICA luminal narrowing. Follow-up brain MRI without contrast on day nine revealed significant interval improvement of the subcortical and deep white matter signal abnormalities (Figure 3).

**Figure 4:**
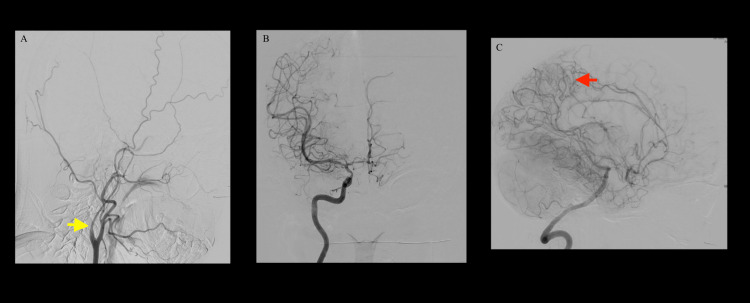
(A-C) Cerebral angiogram performed on day 10 showing (A) a steno-occlusive disease of the left distal ICA (yellow arrow). (B) Selective right ICA injection demonstrated partial opacification of the left anterior cerebral artery via a patent anterior communicating artery. Mild to moderate narrowing of the distal right ICA, MCA, and anterior cerebral artery were observed consistent with intracranial atherosclerotic disease. (C) Selective left vertebral artery demonstrated patent posterior communicating arteries and left posterior cerebral artery-anterior cerebral artery leptomeningeal collaterals (red arrow) contributing to the arterial supply of the left cerebral hemisphere. Internal carotid artery (ICA); middle cerebral artery (MCA)

The following day, a cerebral angiogram demonstrated diffuse narrowing of the left ICA just distal to the carotid bifurcation with no flow of contrast beyond the ophthalmic segment (Figure 4).

During her hospital stay, the patient’s symptoms resolved gradually, and upon discharge on day 12, she had complete resolution of her seizures, hemiparesis, and hemisensory loss and stabilization of her blood pressure readings. The patient was seen in the neurology clinic six months later, and she reported no return of her symptoms, with adequate control of her seizures and blood pressure and a return to her baseline functional status.

## Discussion

This report highlights the neuroradiological features of a rare case of atypical unilateral PRES presenting with headache, left-sided hemiparesis, and sensory loss, with changes in her seizure semiology. We believe that the combination of elevated blood pressure and steno-occlusive disease of the carotid artery results in unilateral PRES of the side contralateral to the steno-occlusive disease.

Unilateral PRES is a rare variant of the syndrome that has been observed in patients with various conditions, including cerebrovascular disease, vascular anomalies, and the use of immunosuppressive and antineoplastic medications [3,6,8,9]. In 2009, McKinney et al. reviewed 76 PRES cases over almost 10 years and described the first two cases of unilateral PRES [1]. The etiology of unilateral PRES remains poorly understood. The literature on unilateral PRES patients suggests that most cases are associated with an underlying vascular anomaly or hemorrhagic irritability [10]. In patients with PRES, an increase in arterial pressure in the cerebral circulation leads to cerebral hyperperfusion that overcomes the autoregulatory capabilities of cerebral blood vessels, resulting in blood-brain barrier disruption, leakage, and vasogenic edema [11]. Chronic steno-occlusive disease of the internal carotid artery results in a reduction in the arterial pressure of arteries distal to the stenosis [12]. This, in turn, could mitigate the effect of high systemic arterial pressure, lead to lower cerebral perfusion in comparison to that of a normal carotid artery, reduce the effect on the blood-brain barrier, and minimize the resultant vasogenic edema. In our case, we propose that unilateral PRES was linked to contralateral steno-occlusive disease of the carotid artery, which potentially ameliorated the effect on the contralateral hemisphere by lowering the perfusion pressure. Similarly, Nishijima et al. reported a patient with a history of left internal carotid artery stenosis and imaging findings consistent with right-sided unilateral PRES, which is similar to the pattern observed in our patient [13]. Dhar et al. reported a patient with subarachnoid hemorrhage (SAH) and vasospasm affecting the right MCA severely and bilateral anterior cerebral arteries moderately, and the imaging findings were consistent with those of left-sided unilateral PRES [10]. One possible explanation is the presence of persistent vasospasm on one side of the cerebral vasculature, resulting in reduced perfusion pressure in that hemisphere. In contrast, the other side of the circulation remains exposed to high mean arterial pressure, making it more susceptible to the development of vasogenic edema [10]. These reports support the evidence that hyperperfusion contributes to pathogenesis. Conversely, there have been reported cases of unilateral PRES without an identifiable cause or association. Ozawa et al. reported a case of unilateral PRES in a patient with a history of lymphoma in remission, chronic hepatitis C, and chronic kidney disease [14]. Similarly, Katsuki et al. described a case of unilateral PRES in a patient with ovarian dysfunction who was receiving cyclical estrogen and progesterone therapy [15]. In both patients, there were no apparent vascular anomalies or cerebrovascular disease contributing to the development of PRES. Hence, further research into this rare variant of PRES is still necessary.

Diagnosing PRES, especially when it is atypical, can be difficult, mainly due to the broad range of potential differential diagnoses. Unilateral PRES may raise suspicion for alternative diagnoses [3,4]. Notably, McKinney et al. reported two cases where radiological findings closely resembled those observed in patients with neoplasms [1]. Fugate et al. proposed diagnostic criteria for PRES, which encompass an acute onset of neurological deficits, focal vasogenic edema, and subsequent resolution of clinical signs, symptoms, and neuroimaging abnormalities [5]. Our patient fulfilled all three criteria. In cases of unilateral involvement, distinguishing between low-grade tumors and PRES can be accomplished by assessing the minimal uptake of methionine in the regions of abnormality on MRI, as well as reduced fluorodeoxyglucose uptake on positron emission tomography scans [16]. Differentiating cytotoxic edema or acute ischemia from vasogenic edema is aided by assessing hyperintense signals on diffusion-weighted imaging and quantifying the apparent diffusion coefficient. Additionally, while bilateral posterior cerebral artery infarctions often involve the paramedian occipital lobes, these regions are typically spared in PRES. Patients with metabolic encephalopathies may exhibit similar clinical presentations to PRES; however, their cerebral imaging findings are usually normal [17].

## Conclusions

Unilateral PRES is a rare variant that can be difficult to diagnose. It is important to consider unilateral PRES in the differential diagnosis of patients with acute neurological symptoms and unilateral vasogenic edema on neuroimaging. We propose that the unilateral PRES in our patient was linked to contralateral steno-occlusive disease of the carotid artery and by lowering the perfusion pressure, mitigating the effect on the contralateral hemisphere. However, further research is warranted to enhance our understanding of its etiology and pathophysiology.
